# Is the dark triad always detrimental to firm performance? Testing different performance outcomes and the moderating effects of competitive rivalry

**DOI:** 10.3389/fpsyg.2023.1061698

**Published:** 2023-03-10

**Authors:** Jarrod Haar, Kirsty de Jong

**Affiliations:** ^1^School of Management, Massey University (Albany), Auckland, New Zealand; ^2^School of Management, Victoria University of Wellington, Wellington, New Zealand

**Keywords:** dark triad, breakthrough sales, firm performance, managerial capital, moderated mediation

## Abstract

There is growing evidence that CEOs who have the ‘dark triad’ of personality traits (Machiavellianism, narcissism, and psychopathy) detrimentally influence firm performance. However, there is still much we do not know. The present study suggests that the CEO dark triad might directly influence typical performance indicators in different ways: positively affecting external performance indicators (breakthrough sales), but negatively affecting internal performance indicators (organizational performance). We argue that the CEO dark triad can be interpreted differently by those external to the firm versus internally, where managers are much closer to the CEO’s dark personality. Our model includes managerial capital as a mediator and competitive rivalry as a moderator, and ultimately tests a moderated mediation model. Using data from 840 New Zealand firms, we find that the dark triad links to outcomes, as expected. While the CEO dark triad is negatively related to managerial capital, managerial capital does positively predict both performance indicators, and partially mediates the CEO dark triad effect. Overall, moderating effects highlight that the CEO dark triad is less detrimental in fiercely competitive business environments, acting as a consistent boundary condition across models. As competitive rivalry increases, the indirect effect of the CEO dark triad on performance decreases. We discuss the implications for understanding the role that the CEO dark triad can play in firms.

## Introduction

Chen et al. (2021) noted the importance of examining CEO narcissism and this is part of the growing attention focusing on unethical behavior (e.g., Chen et al., 2022), including the dark triad (Harrison et al., 2018). This is an important aspect to explore, as CEOs/business leaders guide firms, setting direction and tone. At the CEO level, Peterson et al. (2012) state that “strategic management theory has become increasingly focused on CEOs and their effects on firm-level outcomes” (p. 575). Research focusing on the CEO typically uses the upper echelons theory (UET) (Hambrick and Mason, 1984; Hambrick, 2007), and this has been proven to offer insights into understanding how CEO characteristics, including personality, influence firm performance (Neely et al., 2020). In their meta-analysis of CEO personality and firm performance, Wang et al. (2016) found that UET is the dominant theoretical lens used, and provide evidence that several CEO demographics and personality characteristics shape firm performance. Although the effect sizes are small, some personality factors have greater effects than others, with ‘grandiose self-concept’ aligning strongly with firm risk-taking (corrected mean correlation = 0.18, Wang et al., 2016). The focus on the role of the CEO is relevant given the dramatic changes facing the world regarding technology changes (e.g., Durana et al., 2021; Valaskova et al., 2021) and COVID-19 (e.g., Tijani et al., 2021).

However, while the meta-analysis by Wang et al. (2016) provided strong evidence of CEO’s personality shaping firm performance, there was little exploration of dark personalities, and this is a vital gap that we explore because of their importance to a firm’s ethical behaviors (Chen et al., 2021, 2022). This is important because Islam (2020) called for greater insights into ethical behavior by focusing on personality. Recent attention has begun to examine CEO dark personalities, and while much attention is focused on single traits (e.g., grandiose narcissism, Reina et al., 2014), attention has begun to shift to the broader dark triad because evidence indicates that all three dimensions might occur simultaneously and be especially detrimental. The dark triad represents a collection of three socially undesirable personality traits: Machiavellianism, subclinical narcissism, and subclinical psychopathy, which “despite being undesirable for most concerned, people with higher levels of these traits do not reach disorders clinically” (Bonfá-Araujo et al., 2022, p. 1). They represent non-pathological yet socially aversive traits (Joshanloo, 2021), which “can exist in subclinical levels in normal personality” (McLarnon and Tarraf, 2017, p. 67), and thus represent traits seen within the general population. We acknowledge that there are other ways to explore dark personalities, including the dark tetrad, which is the dark triad plus everyday sadism (Paulhus, 2014), and the dark core, which represents manipulation and callousness (Jones and Figueredo, 2013). Our focus on the dark triad takes established findings on firm performance and then seeks to explore opposite effects.

Jonason and Webster (2010) state that the dark triad “can be thought of as a short-term, agentic, exploitive social strategy that may have evolved to enable exploitation when conspecifics are likely to avoid or punish defectors” (p. 420). One should note that “agentic” here is used from the psychological perspective to describe a preoccupation with performance and status accumulation. McLarnon and Tarraf (2017) state that Machiavellianism “refers to interpersonal behaviors that focus on self-interest, deception, and manipulation of others” (p. 67). Due to this strong self-interest (Jakobwitz and Egan, 2006), Machiavellians are often considered pragmatic, callous, and strategic manipulators (McLarnon and Tarraf, 2017), who act unethically (Harrison et al., 2018). Dahling et al. (2009, p. 227) conceptualized Machiavellianism as “a tendency to distrust others, a willingness to engage in amoral manipulation, a desire to accumulate status for oneself, and a desire to maintain interpersonal control.”

Narcissism relates to internal insecurities and self-grandiose displays (Cesinger et al., 2022), such as presenting “a sense of perceived entitlement and superiority over others” (McLarnon and Tarraf, 2017, p. 68). According to Jakobwitz and Egan (2006), narcissism is often expressed in behaviors such as exhibitionism and constant attention-seeking. In their review of the dark triad, LeBreton et al. (2018) highlighted four common factors: (1) feelings of superiority and a grandiose sense of self, (2) a dysfunctional need for excessive attention and admiration, (3) a propensity to engage in exploitive behaviors, and (4) a lack of empathy. Wisse et al. (2015) noted that narcissists “consider themselves to be superior to others, and strive strongly for power, prestige, and status” (p. 158). The last dimension, psychopathy, reflects being highly impulsive (McDonald et al., 2012), having low empathy (Paulhus and Williams, 2002), and engaging in interpersonal interactions based on arrogance and deceit (Jakobwitz and Egan, 2006). McLarnon and Tarraf (2017) state that psychopathy “reflects individual differences in selfishness, callousness, and superficial charm” (p. 68). LeBreton et al. (2018) suggest psychopathy is arguably the most toxic dimension of the dark triad and summarize it using four key dimensions: interpersonal manipulation (e.g., grandiosity, lying, and superficial charm); callous affect (e.g., lack of empathy and remorse); erratic lifestyle (e.g., impulsivity, irresponsibility, and sensation seeking); and criminal tendencies (e.g., antisocial or counterproductive behavior).

Interestingly, research shows that employees with higher levels of dark triad factors are more likely to become leaders (Brunell et al., 2008). Critically, Smith et al. (2018) argue that the CEO personality literature is too focused on when dark personalities lead to detrimental outcomes, pointing to “emerging evidence [that] suggests that the effects of personality in organizations are far more complex than previously observed” (p. 192). For example, Petrenko et al. (2016) found CEOs’ high narcissism was positively related to the corporate social responsibility (CSR) activities of the firm. That study also highlighted that the CEO dark triad influences on performance are likely to be complex and subject to a range of moderators and mediators (Wang et al., 2016; Palmer et al., 2020; Ahadzadeh et al., 2021). The present study asks three related research questions: (1) what effect does the CEO dark triad have on firm performance? (2) Can it be both detrimental and beneficial to performance? (3) If beneficial, what are the ethical implications for boards that manage such CEOs?

**Figure 1:**
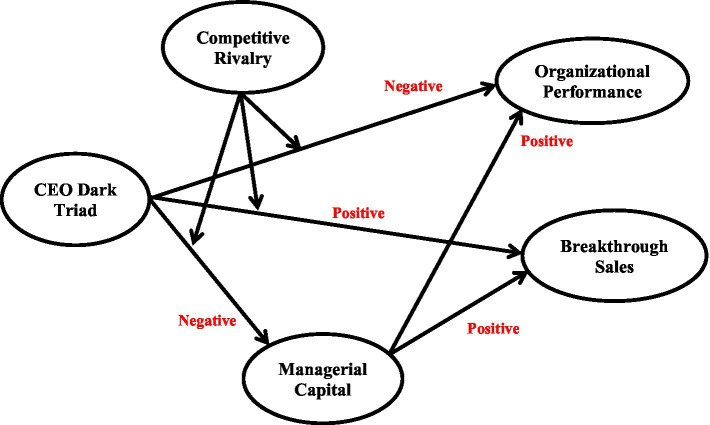
Study model.

Overall, the present study makes three contributions. First, using the logic of Smith et al. (2018), we explore the impact of the CEO dark triad on two performance indicators, exploring potentially both positive and negative effects. Second, evidence suggests that firm performance is shaped by the CEO, including those with the dark triad, as well as top management teams (TMTs) (Engelen et al., 2016; Cesinger et al., 2022). Thus, we test a mediated pathway, whereby the CEO dark triad detrimentally influences managerial capital, which in turn is expected to mediate the CEO’s influence, due to their (theoretical) alignment with TMT (Palmer et al., 2020). Third, we explore competitive rivalry as a moderator because external conditions have been found to influence the impact of CEOs on firm performance. We combine all factors and test a moderated mediation model. Overall, we use a large sample of New Zealand firms to provide insight into our model and to make an important contribution to firm boards that manage CEOs. Our study model is shown in Figure 1.

## Upper echelons theory

Hambrick and Mason (1984, p. 193) introduced the UET to recognize the state of increasing attention given to the characteristics of top management, noting that “the theory states that organizational outcomes, strategic choices, and performance levels are partially predicted by managerial background characteristics,” i.e., managers’ socio-economic information and experiences. The UET has gone on to become “one of the most influential perspectives in management research,” although not without critique (Neely et al., 2020, p. 1,029). Li et al. (2015) argued that top managers play critical roles in decision-making, such as resource allocation, and thus “the collective characteristics of top executives guide their strategic choices, which influence their organizations” (p. 941). According to Waldman et al. (2001), the UET suggests that specific characteristics and the leadership of top managers ultimately shape firm performance, but argued against focusing only on demographics (e.g., experience), stating that “a consideration of personal or leadership characteristics is necessary for a more complete test of upper echelons theory” (p. 134).

In their study, Peterson et al. (2012) found support for UET combined with executive personality (e.g., Peterson et al., 2009), providing evidence that senior executives’ leadership behaviors are shaped by their backgrounds and personalities. For example, CEO narcissism is positively related to founder status, but this is oppositely related to servant leadership, which was positively related to performance in technology firms. A recent review and meta-analysis by Cragun et al. (2020) noted that the majority of CEO dark personality research, which heavily favors narcissism, “is explored through the lens of upper echelons theory and leadership theory. Upper echelons theory is a logical and appropriate framing for CEO narcissism research because it connects the CEO’s motivations and attributes with organizational outcomes” (p. 926). As a caveat, the authors suggest that while the UET approach has been fruitful for understanding CEOs and TMTs, the framework is somewhat limited, often failing to examine the business environment firms operate in.

## The dark triad

Paulhus and Williams (2002: 557) note that the dark triad dimensions have distinct origins, but share several features: “To varying degrees, all three entail a socially malevolent character with behavior tendencies toward self-promotion, emotional coldness, duplicity, and aggressiveness.” A meta-analysis at the employee level (O’Boyle et al., 2012) found that Machiavellianism and psychopathy were negatively related to job performance, but with small effect sizes. However, all three dimensions of the dark triad were positively related to counterproductive work behaviors, and accounted for moderate amounts of variance. Wisse et al. (2015) argued that the dark triad is especially relevant to the study of leaders because, while these traits are accepted as generally socially undesirable, in an organizational context, the dark triad might be beneficial. For example, Zettler et al. (2010) found that Machiavellianism was positively related to employee career commitment.

Despite growing attention to components of the dark triad such as narcissism (e.g., Chen et al., 2021), we understand little about the dark triad in terms of leadership and firm performance in New Zealand. LeBreton et al. (2018) noted that a leader’s dark triad can influence outcomes, including personal subordinates’ job satisfaction. Regarding narcissism, employees with high scores are not only more likely to become leaders but are also subsequently rated as more effective leaders (Brunell et al., 2008). Finally, psychopathy is positively linked to charisma and leaders’ presentation style (Babiak et al., 2010). Despite acknowledgments in the literature that CEOs and TMTs help shape firm performance and vital factors such as entrepreneurship (Engelen et al., 2016; Cesinger et al., 2022), there are still gaps in our understanding of the influence of the dark triad on firms’ performance.

Recent studies on firm performance have included leader dominance and self-esteem as positive influences (Palmer et al., 2019), while Kraus et al. (2018) examined the effect of the dark triad on firm performance, finding significant correlations but no significant direct or moderation effects. Palmer et al. (2020) noted that, while there has been a lot of focus on executive traits and the dark triad, empirical tests of the dark triad as a collective have been scant. In their meta-analysis of CEO narcissism, Cragun et al. (2020) reported significant but small effects on some performance indicators but not others, concluding mixed findings. Zhang et al. (2017) explored CEO narcissism and found that it was not related to either firm innovation or firm performance, although it did interact significantly with CEO humility, leading to higher firm innovation. Overall, Palmer et al. (2020) theorized that the influence of a CEO’s dark triad on firm performance might be best explained as a process whereby CEO traits shape interactions with the TMT, and this subsequently shapes subordinates’ behavior and performance.

Finally, Smith et al. (2018) argues that dark personality traits (e.g., the dark triad) might not be universally detrimental, and, indeed, may exert interesting effects. For example, Wales et al. (2013) found that CEO narcissism was positively related to firm performance and entrepreneurial orientation. Smith et al. (2018) suggest that dark personalities might be more effective in certain roles and situations, highlighting that while “certain image-enhancing traits like narcissism and Machiavellianism may be beneficial for climbing the corporate ladder, these traits also appear to benefit external stakeholders” (p. 206) in the way that they perceive a firm *via* the CEO. The authors also argued that researchers need to explore why and where dark traits might be conducive to good firm outcomes, suggesting that narcissists have a higher need for external approval, which might drive creativity. This is due to the importance of creativity (Wang et al., 2022).

The present study explores the potential positive links between the CEO dark triad and firm performance. Our first performance indicator is breakthrough sales, which refers to the percentage of total sales generated from new products (Hall and Mairesse, 2006). We take breakthrough sales as our external performance indicator and hypothesize beneficial effects from bad behavior stemming from the CEO’s dark triad. This is counter to the expected detrimental performance effects, at least at the individual level (O’Boyle et al., 2012). While CEO personality meta-analytically supports firm performance, the links between performance and the dark triad are lacking (Wang et al., 2016). Here, we argue for positive effects because this aligns with the arguments made by Smith et al. (2018) for testing beneficial and not only detrimental effects. We suggest that the collective impact of the desire to accumulate status (Machiavellianism), coupled with grandiose goals and the need for excessive attention and admiration (narcissism), in combination with grandiosity and sensation-seeking erraticism (psychopathy), will benefit (not harm) breakthrough sales. Here, we suggest that this combination could manifest as greater “show-person” behaviors from the CEO that raise a firm’s profile, improving its chances of capturing new sales. Indeed, given the nature of these behaviors, breakthrough sales—entering new markets, obtaining new customers, and conquering new places—align theoretically well with the CEO’s dark triad and the potential for beneficial effects. Hence, we expect that breakthrough sales will benefit from the CEO dark triad.

*Hypothesis 1:* The CEO dark triad will be positively related to breakthrough sales.

Our other performance indicator is organizational performance, which is distinct from breakthrough sales. Here, we take a more internal view of the firm—distinct from external sales—and focus on employee factors, including job satisfaction and retention, as well as workforce skills and service, and the effectiveness of workforce leaders such as supervisors (Yang and Lin, 2009). This measure is distinct from sales, where the public persona of a CEO with high levels of the dark triad might otherwise be more easily controlled and manipulated. These factors—job satisfaction, workforce retention, and workforce skills—all meta-analytically support firm performance (Judge et al., 2001; Crook et al., 2011; Park and Shaw, 2013). We argue that the same behaviors noted above (e.g., grandiose behaviors, attention-seeking, etc.) will be detrimental to organizational performance because the workforce will be able to see different sides of the dark triad beyond the public role of positively “selling the firm”. For example, managers (the target respondents of this study) might be more inclined to have experienced the deception, manipulation, and callous, unethical behavior of their CEO (Machiavellianism); to have noted their sense of entitlement and superiority (narcissism); and to have witnessed the deceit, arrogance, and low empathy (psychopathy) of a dark triad CEO. Thus, we expect the CEO dark triad to be more apparent to managers and their workforce, thereby detrimentally affecting organizational performance. Consequently, we suggest that the CEO dark triad will be negatively related to organizational performance. We posit the following:

*Hypothesis 2:* The CEO dark triad will be negatively related to organizational performance.

## Managerial capital

Smith et al. (2018) suggested that the links between firm performance and CEO dark traits might be complex. Reina et al. (2014) highlighted mediating mechanisms through which CEOs influence firm performance, and this mediation call was reiterated in a CEO narcissism meta-analysis and review (Cragun et al., 2020). We draw on the theoretical model of Palmer et al. (2020) that argued TMTs are likely to play a key role; we thus use managerial capital as a mediator. Here, managerial capital refers to the structural elements of an organization capturing the knowledge and experience of the TMT and the way the TMT facilitates a workforce’s ability to create firm value (Yang and Lin, 2009). Bontis (1999) suggests that this includes knowledge access, efficiency, and innovativeness. Thus, a strong managerial capital represents a firm with a powerful TMT that creates processes that aid knowledge sharing, efficiency, and innovativeness, and generates superior performance through greater firm efficiency and customer capture (Yang and Lin, 2009). Considering the empirical model of Reina et al. (2014), we focus on the TMT because they reflect the high-level management processes of the firm (Yang and Lin, 2009), which, in turn, are contingent on the CEO’s leadership style. Ultimately, this form of capital (TMT) represents firm resources reflective of managerial skills (Subramaniam and Youndt, 2005).

Zollo and Winter (2002) argue that strong managerial capital ensures that a firm can maximize organizational resources, such as in leveraging acquired knowledge. Empirical evidence provides support for the fact that intellectual capital plays a valuable role in firm performance (e.g., Yang and Lin, 2009). In their meta-analysis, Albertini and Berger-Remy (2019) reported a moderate effect size of intellectual capital on accounting-based indicators (0.19) and overall financial performance (0.20). While the UET sometimes focuses on TMT (Carpenter, 2002), meta-analytic evidence (Certo et al., 2006) suggests only a small effect from TMT demographic factors (e.g., position tenure). Here, we focus not on TMT demographics but instead on the broad representation of the managerial capital of a firm. We suggest that the managerial capital of a firm, representing the top managers apart from the CEO, should play a key role in firm performance. This aligns with the UET approach, and, based on meta-analytic support (Albertini and Berger-Remy, 2019), we expect managerial capital to shape performance. Thus, firms with strong managerial capital are expected to have superior processes and pay greater attention to knowledge usage, efficiency, and customers. This aligns well with increased breakthrough sales and makes the firm a better place to work, reflecting enhanced organizational performance. Further, we expect managerial capital to be negatively influenced by the CEO’s dark triad—reflecting the detrimental effects noted above—we expect the TMT to be especially exposed to the CEO’s dark side (deception, manipulation, superiority, deceit, and low empathy). This is likely to lead to issues related to disrupting focus time and undermining the efficiencies of processes and knowledge sharing (e.g., Haar et al., 2022a). Hence, we expect the CEO dark triad to be detrimental to managerial capital. We posit the following:

*Hypothesis 3:* The CEO dark triad will be negatively related to managerial capital.

*Hypothesis 4:* Managerial capital will be positively related to (a) breakthrough sales and (b) organizational performance.

Finally, we also expect managerial capital to mediate the effects of CEO personality, aligning with recent arguments (Reina et al., 2014; Smith et al., 2018; Palmer et al., 2020). Thus, the influence of a CEO’s dark triad is better explained by reducing the effectiveness of managerial capital, which, in turn, is expected to be positively related to both breakthrough sales and organizational performance. However, we do not hypothesize a mediation effect from managerial capital toward the relationship between the CEO dark triad and breakthrough sales. Here, we argue that the positive and beneficial effect of the CEO dark triad comes directly from the persona the dark triad CEO can project. While managerial capital is still expected to be positively related to breakthrough sales, we do not suggest that managerial capital will mediate the CEO’s dark triad. Again, the external focus on breakthrough sales and the associated desire to accumulate status (Machiavellianism), the grandiose goals and the attention and admiration needs due to narcissism, and sensation-seeking erraticism (psychopathy) will ultimately occur regardless of the managerial capital processes and efficiency. Thus, we hypothesize a mediation effect toward organizational performance only. We posit the following:

*Hypothesis 5:* Managerial capital will mediate the influence of the CEO’s dark triad on organizational performance.

## Competitive rivalry

While the dark triad literature calls for mediation testing, it has also identified moderators as a key aspect that require more attention (e.g., Reina et al., 2014; Smith et al., 2018; Palmer et al., 2020). For example, Engelen et al. (2016) found that narcissistic CEOs diluted the impact of entrepreneurial orientation on firm performance, although the effect was positive in highly dynamic (i.e., competitive) markets. Theoretically, with UET, we know that top managers guide strategic choices, including resource allocation (Li et al., 2015), with Hambrick and Mason (1984) noting that UET allows us to understand why organizations act as they do. Here, we include the role of competitive rivalry as a moderator because it captures the context that firms operate in, ranging from highly competitive environments to benign and placid environments. Competitive rivalry represents an evaluation of the extent and intensity of competition facing a firm (Spanos and Lioukas, 2001), with Porter (2008) arguing that competitive rivalry “reflects not just the intensity of competition but also the basis of competition” (p. 32), for example, a dominant competitor that might impede a firm’s ability to make a profit. The latter aligns well with our focus on firm performance, and captures why TMTs react to competitive rivalry—because to ignore the context of the business environment is to invite financial loss. Aligned with TMTs, Reina et al. (2014) identified that the competitive landscape might moderate their influence. This is because a TMT includes those who, under UET, make decisions based on additional external pressures on their firms. Waldman et al. (2001) found evidence that environmental uncertainty interacts with CEO characteristics (charisma) in terms of net profit. Thus, we understand that the external environment can influence the way CEOs affect firm performance; this should similarly align with the CEO dark triad. Overall, these studies encourage testing of the external environment as a moderator.

Fouskas and Drossos (2010) stated that business environments that are fiercely competitive means competitor actions “are more visible and threatening” and “firms are expected to respond aggressively in order to maintain their market share” (p. 481). There is strong evidence that competitive rivalry helps shape firm performance (e.g., Gilman et al., 2015). Overall, we expect the influence of the CEO dark triad to interact favorably when competitive rivalry levels are high (representing fierce competition), although influencing firm factors and performance somewhat differently. We suggest that CEOs with high levels of the dark triad might effectively be “in their element”, where they face new attention from competitors and stakeholders, potentially enjoying the attention due to their psychopathy. Thus, we expect higher breakthrough sales. However, in terms of managerial capital and organizational performance, which are both expected to be negatively influenced by the CEO dark triad, we expect the detrimental influence of the CEO to be reduced (i.e., a weaker negative effect) when firms operate in more competitive environments. In effect, competitive rivalry in the business environment can buffer the otherwise detrimental effects of a CEO that has a high level of the dark triad on their firm. This likely brings aspects of the dark triad positively to bear, such as the firm gaining more attention from the actions of the CEO.

Beyond direct effects and two-way interactions, competitive rivalry is also explored as a boundary condition (Hayes, 2018), which represents an analytical strategy focused on “the contingent nature of processes, meaning whether “mediation is moderated”” (p. 2). Thus, the indirect effect of the CEO dark triad on performance indicators (*via* managerial capital as the mediator) will be dependent on the moderator (competitive rivalry). Here, we expect that the indirect effect of the CEO dark triad will become weaker as competitive pressures rise, with the influence of managerial capital becoming more important (Albertini and Berger-Remy, 2019). We posit the following:

*Hypothesis 6:* Competitive rivalry will be positively related to (a) breakthrough sales and (b) organizational performance.

*Hypothesis 7:* Competitive rivalry will interact with the CEO’s dark triad in terms of (a) managerial capital, (b) breakthrough sales, and (c) organizational performance, leading to stronger beneficial effects and weaker detrimental effects.

*Hypothesis 8:* The indirect relationship between the CEO dark triad and (a) breakthrough sales and (b) organizational performance, via managerial capital, will be moderated by competitive rivalry. The indirect effects become weaker as rivalry increases (moderated mediation).

## Methods

### Participants and sample

A total of 840 participants were recruited early in 2020 (pre-COVID-19 events) *via* a Qualtrics survey panel of New Zealand firms. An initial screening question confirmed that respondents were in a senior management role (e.g., executive/senior manager/manager) and were adequately able to reflect on their firm’s activities. CEOs were removed from the study. Researchers can copy this methodology by purchasing firm data through a similar panel, seeking a broad range of manager respondents (all non-CEO) across a representative sample of firms within their country. Qualtrics ensures quality respondents (removing those who answer too fast or too slow and single response only), and this methodological approach has yielded positive samples (e.g., Haar et al., 2022b). Overall, all senior leaders in their panel database were targeted, and they came from firms across all industries. From the database, a rolling sample of potential respondents were contacted *via* email (with the survey link), and the number of leaders contacted was increased until the target quota was achieved (here *n* = 840 respondents). Then, data collection was stopped. All participants answered all questions and thus were included in the study. They represent 840 senior managers from unique firms.

A meta-analytic comparison found that such panel data did not significantly differ from conventional data (Walter et al., 2019). While we utilized single-sourced data, we acknowledge issues highlighted by Podsakoff et al. (2003) regarding common method bias (CMB); we ensured that our study factors were separated by unrelated questions to ensure respondents were not only discussing their firm, but also their personal experiences in between constructs on their firm.

Respondents had an average tenure in their current job of 7.79 years (SD = 5.7). Firms had an average age of 34.2 years (SD = 31.1) and were well spread by size: 24.4% were micro-sized (up to 10 employees), 25.1% were small-sized (11–50 employees), 26.0% were medium-sized (51–250 employees), and 24.5% were large-sized (251 or more employees). The average level of workplace education was similarly well spread: 24.3% had high school qualifications, 23.8% had technical college qualifications, 39.3% had bachelor’s degrees, and 12.6% had postgraduate qualifications. Finally, respondents came from a large number of different industries, with the largest being manufacturing (10.2%), retail (9.6%), education and training (9.0%), and professional services (8.9%). Our focus was on trying to gain generalizability of effects; thus, we had a wide industry focus.

#### Measures

The CEO dark triad was measured using the ‘dirty dozen’: a 12-item short measure by Jonason and Webster (2010), coded 1 = strongly disagree through to 5 = strongly agree. We followed the approach of Mutschmann et al. (2022) and had managers rate their leaders, in this case, CEOs. All items were focused on the firm’s leader, specifically “My CEO/Top Manager....” The sample items used were: “Has used deceit or lied to get their way” (Machiavellianism); “Tends to want others to pay attention to them” (narcissism); and “Tends to be unconcerned with the morality of his/her actions” (psychopathy). We conducted confirmatory factor analysis (CFA) in AMOS (version 25), using the guidelines set out in the literature (e.g., Williams et al., 2009) on three goodness-of-fit indices: (1) the comparative fit index (CFI ≥0.90), (2) the root-mean-square error of approximation (RMSEA ≤0.08), and (3) the standardized root mean residual (SRMR ≤0.10). We tested a higher-order model with the three dimensions loading onto a single factor (CEO dark triad), and this demonstrated a good fit to the data: *χ*^2^(df) = 378.6(53), CFI = 0.97, RMSEA = 0.08, and SRMR = 0.03. The combined construct had excellent reliability (*α* = 0.97).

Organizational performance was measured using four items from Yang and Lin (2009), coded 1 = strongly disagree through to 5 = strongly agree. Questions followed the stem “To what extent does your organization engage in the following,” and sample items include “Our manager and supervisors are effective” and “Our employees have high job satisfaction” (*α* = 0.77).

Breakthrough sales were used as an indicator of innovation performance following Faems et al. (2005), and were captured with a single item. Often, breakthrough sales are thought about in terms of the proportion of turnover of stock of a new product for a given period, such as in the last 3–5 years (Faems et al., 2005; de Visser et al., 2010). This approach has been used by others to assess the impact of firm structure on innovation performance (e.g., de Visser et al., 2010). Using the wording from Laursen and Salter (2006), with the stem “Within the last 3 years…” we asked, “What percentage of overall organization sales/income comes from products and services new to your organization?” (response scale ranged from 0 to 100%).

Managerial capital was measured using the 3 items in Yang and Lin (2009), coded 1 = strongly disagree through to 5 = strongly agree. We shaped items to align with the top-management team focus used by Palmer et al. (2020). Sample items included “Our top management team regards employees as the source of value creation” and “Our organization has an effective top management process” (*α* = 0.83).

#### Control variables

We controlled for firm age (in years) and firm size 1 = micro-sized (up to 10 employees), 2 = small-sized (11–50 employees), 3 = medium-sized (51–250 employees), and 4 = large-sized (251 or more employees). We expect older firms to be more established and larger-sized firms to have greater resources that might shape performance (e.g., Gibb and Haar, 2010). We also controlled for the private sector (1 = yes and 0 = no) and family business (1 = yes and 0 = no), as these might affect the performance indicators (e.g., Lee, 2006). Finally, because our sample included 20 industries, we controlled for industries most likely to influence performance: manufacturing, professional services, education and training, information, media and telecommunications (info-tech), and healthcare and social assistance (healthcare).

#### Measurement models

We confirmed the distinct nature of the various study constructs using CFA in SEM with AMOS version 26, following standard thresholds (Williams et al., 2009). We tested alternative CFAs to determine if the theoretically derived constructs best fit the data shown in Table 1.

**Table 1:** Results of confirmatory factor analysis.

	Model fit indices				Model differences				
Model	*χ* ^2^	df	CFI	RMSEA	SRMR	*χ* ^2^	Δdf	*p*	Details
Model 1	711.4	183	0.96	0.06	0.04				
									
Model 2	2035.4	186	0.86	0.11	0.26	1324.0	3	0.001	Model 1 to 2
									
Model 3	818.6	185	0.95	0.06	0.07	107.2	2	0.001	Model 1 to 3

Model 1 = hypothesized 4-factor model: dark triad (higher order model of Machiavellianism, narcissism, and psychopathy), managerial capital, firm performance, and breakthrough sales.

Model 2 = alternative 3-factor model: same as model 1 but with dark triad and managerial capital combined.

Model 3 = alternative 3-factor model: same as model 1 but with firm performance and breakthrough sales combined.

Overall, the hypothesized measurement model was the best fit for the data: *χ*^2^(df) = 711.4(183), CFI = 0.96, RMSEA = 0.06, and SRMR = 0.04. Alternative measurement models resulted in a poorer fit (all *p* values<0.001) compared with the hypothesized model (Hair et al., 2010).

#### Analysis

Hypotheses were tested in SPSS (version 25) using the PROCESS 3.4 program (Hayes, 2018). We used model 4 to test mediation and model 8 to test for moderation and moderated mediation effects. We followed the recommendations made by Hayes (2018) regarding mediation tests, and tested indirect effects *via* bootstrapping (5,000 times). Confidence intervals (CI) are reported as lower limits (LL) and upper limits (UL) at the 95% level. We explored the data for outliers and five were identified for firm size (being the largest) and two for age. The latter group of two firms was also in the first group. We tested models excluding and including these potential outliers; the effects remained the same; therefore, the entire sample was ultimately retained. Overall, data were normally distributed.

## Results

Descriptive statistics for the study variables are shown in Table 2, using Pearson correlations, with the assumption that data are interval scoring and normally distributed (which they are).

**Table 2:** Correlations and descriptive statistics of study variables.

Variable	Mean	SD	1	2	3	4	5	6	7
1. Firm age	34.2	31.1	–						
2. Firm size	2.51	1.11	0.37**	–					
3. Dark triad	2.57	1.1	−0.03	0.21**	–				
4. Managerial capital	3.72	0.84	−0.11**	−0.10**	−0.21**	–			
5. Competitive rivalry	3.33	0.66	−0.01	0.14**	0.08*	0.25**	–		
6. Organizational performance	3.76	0.65	−0.13**	−0.22**	−0.25**	0.66**	0.23**	–	
7. Breakthrough sales	22.9	27.8	0.14**	−0.12**	0.28**	0.17**	0.30**	0.12**	–

*N* = 840, *p < 0.05, **p < 0.01.

Table 2 shows that the CEO dark triad is significantly correlated with managerial capital (*r* = −0.21, *p* < 0.01), competitive rivalry (*r* = 0.08, *p* = 0.017), organizational performance (*r* = −0.25, *p* < 0.01), and breakthrough sales (*r* = 0.28, *p* < 0.01). Managerial capital is significantly correlated with competitive rivalry (*r* = 0.25, *p* = 0.017), organizational performance (*r* = 0.66, *p* < 0.01), and breakthrough sales (*r* = 0.17, *p* < 0.01). Competitive rivalry is significantly correlated with organizational performance (*r* = 0.23, *p* < 0.01) and breakthrough sales (*r* = 0.30, *p* < 0.01), and the two performance indicators are only modestly correlated (*r* = 0.12, *p* < 0.01). Importantly, this analysis confirms that the CEO dark triad is significantly correlated with both firm performance indicators, in opposite directions.

**Figure 2:**
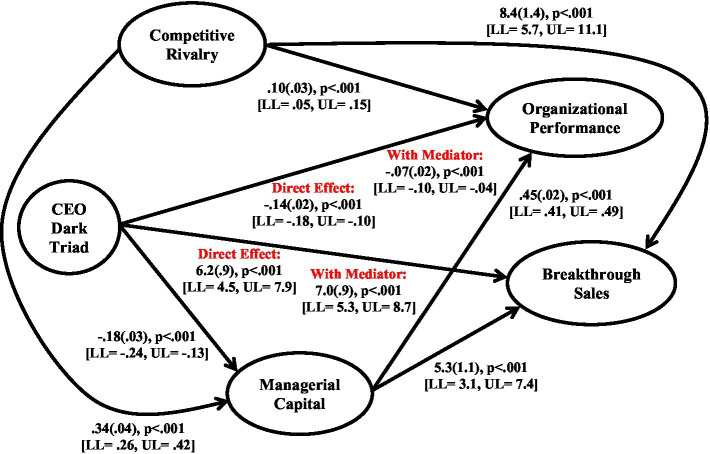
Summary of direct and mediation effects.

The results of the direct and mediation analyses of firm performance indicators are shown in Figure 2.

The results show that the CEO dark triad is significantly related to managerial capital (*β* = −0.18(0.03), *p* < 0.001 [LL = −0.24, UL = −0.13]), organizational performance (*β* = −0.14(0.02), *p* < 0.001 [LL = −0.18, UL = −0.10]), and breakthrough sales (*β* = 6.2(0.9), *p* < 0.001 [LL = 4.5, UL = 7.9]), supporting Hypotheses 1–3. Managerial capital is significantly related to organizational performance (*β* = 0.45(0.02), *p* < 0.001 [LL = 0.41, UL = 0.49]) and breakthrough sales (*β* = 5.3(1.1), *p* < 0.001 [LL = 3.1, UL = 7.4]), supporting Hypotheses 4a and 4b. The inclusion of managerial capital in the models partially mediates the effect of the CEO dark triad on organizational performance, with the direct effect decreasing (*β* = −0.07(0.02), *p* < 0.001 [LL = −0.10, UL = −0.04], although the indirect effect remains significant. In terms of breakthrough sales, there is a slight increase (not decrease) in the direct effect of the CEO dark triad (*β* = 7.0(0.9), *p* < 0.001 [LL = 5.3, UL = 8.7]). This provides support for the mediation argument regarding organizational performance (Hypothesis 5) and confirms that there is no mediation effect on breakthrough sales.

Regarding the direct effect of competitive rivalry, this was significantly related to managerial capital (*β* = 0.34(0.04), *p* < 0.001 [LL = 0.26, UL = 0.42]), organizational performance (*β* = 0.10(0.03), *p* < 0.001 [LL = 0.05, UL = 0.15]), and breakthrough sales (*β* = 8.4(1.4), *p* < 0.001 [LL = 5.7, UL = 11.1]), supporting Hypotheses 6a–6c.

The results of the moderation and moderated mediation analysis in terms of firm performance indicators are shown in Table 3.

**Table 3:** Results of moderated regression analyses.

Variable	Β (SE)	Confidence interval	*p*-value
*Controls:*			
Workforce education ➔ managerial capital	−0.22 (0.10)	LL = −0.42, UL = −0.02	*p* = 0.030
Firm age ➔ managerial capital	−0.003 (0.001)	LL = −0.005, UL = −0.001	*p* = 0.004
Firm size ➔ performance	−0.08 (0.02)	LL = −0.12, UL = −0.05	*p* < 0.001
Professional services ➔ performance	0.12 (0.06)	LL = 0.01, UL = 0.24	*p* = 0.036
Firm size ➔ sales	−0.11 (0.03)	LL = −0.18, UL = −0.05	*p* < 0.001
Firm age ➔ sales	3.2 (1.1)	LL = 1.4, UL = 5.0	*p* < 0.001
Manufacturing ➔ sales	6.7 (3.0)	LL = 0.92, UL = 12.5	*p* = 0.023
			
*Interactions:*			
Dark triad x comp. Rival. ➔ managerial capital	0.10 (0.03)	LL = 0.03, UL = 0.16	*p* = 0.005
Dark triad x comp. Rival. ➔ performance	0.06 (0.02)	LL = 0.02, UL = 0.10	*p* = 0.002
Dark triad x comp. Rival. ➔ sales	1.4 (1.1)	LL = −0.75, UL = 3.5	*p* = 0.201
			
*Index of Moderated Mediation:*			
Dark triad ➔ managerial capital ➔ performance (x comp. Rival.)	0.04 (0.02)	LL = 0.01, UL = 0.08	*p* = 0.008
Dark triad ➔ managerial capital ➔ sales (x comp. Rival.)	0.51 (0.24)	LL = 0.06, UL = 1.0	*p* = 0.016
			
Total *R*^2^ managerial capital	0.15	*F* score = 11.781 (*p* < 0.001)	
Total *R*^2^ performance	0.49	*F* score = 60.473 (*p* < 0.001)	
Total *R*^2^ sales	0.22	*F* score = 17.669 (*p* < 0.001)	

β, unstandardized regression coefficients; SE, standard error. Confidence intervals are 95% and LL, lower limit, UL, upper limit. All significance tests were two-tailed.

Comp. rival., competitive rivalry; performance, firm performance; sales, breakthrough sales. Only significant control variables are shown.

**Figure 3:**
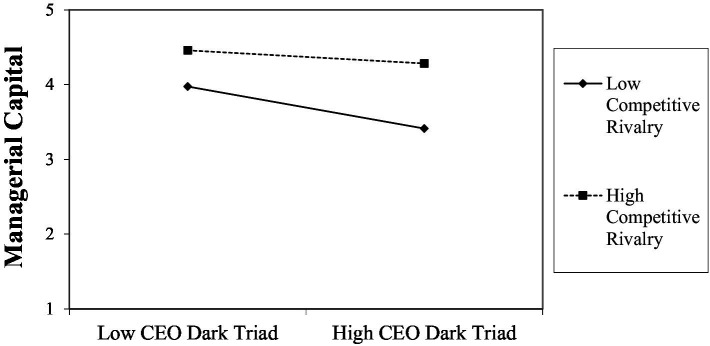
Interaction effects of competitive rivalry on CEO dark triad in terms of managerial capital.

**Figure 4:**
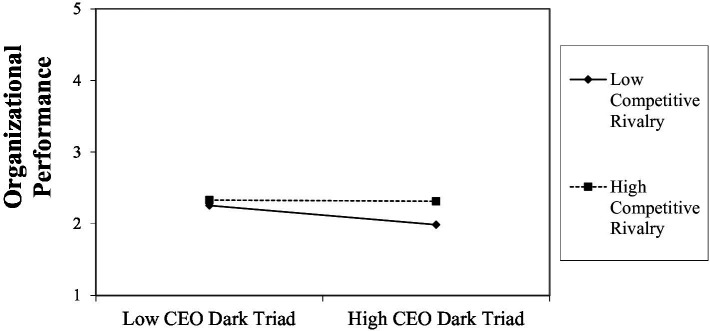
Interaction effects of competitive rivalry on CEO dark triad in terms of organizational performance.

**Figure 5:**
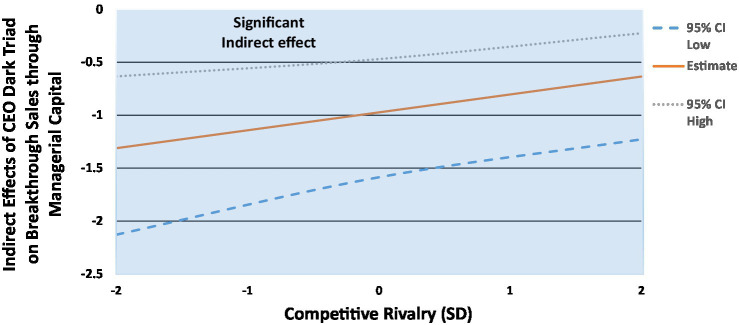
Indirect effects of CEO dark triad on breakthrough sales through managerial capital, conditional on competitive rivalry.

**Figure 6:**
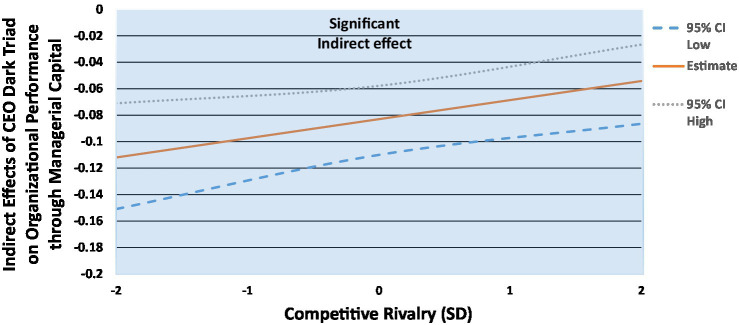
Indirect effects of CEO dark triad on organizational performance through managerial capital, conditional on competitive rivalry.

Significant interactions were found between the CEO dark triad and competitive rivalry in terms of managerial capital (*β* = 0.10(0.03), *p* = 0.005 [LL = 0.03, UL = 0.16]) and organizational performance (*β* = 0.06(0.02), *p* = 0.002 [LL = 0.02, UL = 0.10], but not breakthrough sales (*β* = 1.4(1.1), *p* = 0.201 [LL = −0.75, UL = 3.5]). This supports Hypotheses 7a and 7c. Finally, regarding the moderated mediation (Hypotheses 8), a significant indication of moderated mediation was found in terms of competitive rivalry on the indirect effect of the CEO dark triad on breakthrough sales, with managerial capital (*index* = 0.51(0.24), *p* = 0.016, LLCI = 0.06, ULCI = 1.0) and organizational performance (*index* = 0.04(0.02), *p* = 0.008, LLCI = 0.01, ULCI = 0.08) mediating. This supports both Hypotheses 8a and 8b. We graphed the interaction to illustrate the effects, and these graphs are shown in Figures 3–6.

The interaction effects toward managerial capital (Figure 3) show that at low levels of the CEO dark triad, there is a significant difference in the levels of managerial capital, with firms in fiercely competitive environments reporting higher levels of managerial capital compared with firms in weak competitive rivalry markets. When compared with firms with high levels of the CEO dark triad, firms in fiercely competitive environments report similarly high levels of organizational capital, while those in weak competitive environments report a significant drop in organizational capital. This supports Hypothesis 7a regarding fierce competitive rivalry minimizing the negative effect of the CEO dark triad.

Figure 4 shows the interaction effects toward organizational performance and shows that at low levels of the CEO dark triad, there is no significant difference in levels of organizational performance for firms in either fierce or weak competitive environments. When compared with firms with high levels of the CEO dark triad, firms operating in fiercely competitive environments report stable and similarly higher levels of organizational performance, while firms operating in weak competitive markets report a significant drop in organizational performance. This supports Hypothesis 7c regarding fierce competitive rivalry minimizing the negative effect of the CEO dark triad.

Figures 5, 6 show the significant moderated mediation effects on breakthrough sales (Figure 5) and organizational performance (Figure 6), and we follow Wayne et al. (2017) and probe the conditional indirect effects of the CEO dark triad on performance through managerial capital, conditional on the magnitude of competitive rivalry (effects at −2SD, mean, and + 2SD). In terms of breakthrough sales, we find that in weak competitive rivalry settings (−2SD), the indirect effect of the CEO dark triad on breakthrough sales vis-à-vis managerial capital is significant and negative (*β* = −1.3(0.39), *p* < 0.001; LLCI = −2.1; ULCI = −0.63), and, at average competitive rivalry (mean), the indirect effect is significant and negative but weaker (*β* = −0.97(0.29), *p* < 0.001; LLCI = −1.6; ULCI = −0.47). Finally, when competitive rivalry is fierce (+2SD), the indirect effect of the CEO dark triad is still significant and negative but drops in strength again (*β* = −0.63(0.25), *p* = 0.006; LLCI = −1.2; ULCI = −0.23). While the indirect effect is significant across the full 95% confidence intervals, the indirect effect of the CEO dark triad on breakthrough sales decreases as firms report stronger competitive rivalry, providing support for Hypothesis 8a.

Regarding organizational performance, we find that in weak competitive rivalry settings (−2SD), the indirect effect of the CEO dark triad on organizational performance vis-à-vis managerial capital is significant and negative (*β* = −0.11(0.02), *p* < 0.001; LLCI = −0.15; ULCI = −0.07), and at average competitive rivalry (mean), the indirect effect is significant and negative but weaker (*β* = −0.08(0.01), *p* < 0.001; LLCI = −0.11; ULCI = −0.06). Finally, when competitive rivalry is fierce (+2SD), the indirect effect of the CEO dark triad is still significant and negative, but weaker again (*β* = −0.05(0.02), *p* < 0.001; LLCI = −0.09; ULCI = −0.03). While the indirect effect is significant across the full 95% confidence intervals, the indirect effect of the CEO dark triad on organizational performance decreases as firms report stronger competitive rivalry, again supporting Hypothesis 8b.

Finally, the control variables show that in terms of managerial capital, both workforce education and firm age are significant and negatively related. Regarding organizational performance, significant effects are found from firm size (negative) and professional services (positive). In terms of breakthrough sales, firm size is significantly and negatively related, while firm age and manufacturing are significantly and positively related. Overall, the models are all significant (p < 0.001) and account for robust but modest levels of variance for managerial capital (15%) and breakthrough sales (22%), but large amounts of variance for organization performance (49%).

## Discussion

The study of the effects of CEO personality on firm performance is still in its infancy, and scholars have urged for more testing. Further, researchers have also called for the incorporation of mediation and moderation effects (e.g., Reina et al., 2014; Smith et al., 2018; Palmer et al., 2020) and to expand the focus beyond the assumption that dark personalities are always detrimental (Smith et al., 2018). The present study explored the CEO dark triad, representing Machiavellianism, narcissism, and psychopathy, and it is the collection of all of these three traits that can shape the potential “dark side” of leadership. Further, while the CEO dark triad provides meta-analytic support for the largely detrimental effects at the employee level (e.g., counterproductive work behaviors, O’Boyle et al., 2012), we argued that detrimental effects were more likely to affect organizational performance and managerial capital. This captures the internal workings of a firm (performance) and top management (managerial capital), and these employees and leaders are more likely to witness CEOs with high levels of the dark triad behaving poorly. Indeed, our hypothesized detrimental direct effects of the CEO dark triad were supported. Hence, regarding organizational performance and managerial capital, our findings align with the meta-analysis (Smith et al., 2018).

Our other direct effect hypothesis followed arguments made by Smith et al. (2018), who suggested that dark personalities might conceivably be beneficial. We argued that breakthrough sales (representing new sales from new products/markets) could be positively shaped by the CEO’s dark triad, and this was supported. We suggested that the typically offensive personality might be potentially appealing for public display, with aspects such as grandiose and attention-seeking behavior, perhaps in combination with a sensation-seeking erratic lifestyle, attracting new customers and shaping breakthrough sales. Our findings support Smith et al. (2018), who argued that certain image-enhancing traits (e.g., narcissism and Machiavellianism) are beneficial for external stakeholders because they are constructed with others in mind. Overall, we find that the CEO dark triad is both detrimental and beneficial to firm performance, specifically those constructs targeting internal and external indicators. We suggest that this finding provides insights into why CEOs with high levels of the dark triad maintain employment and find new employment when they do shift jobs. Ultimately, their personality does have benefits for firms, at least regarding external stakeholders (e.g., customers) rather than internal stakeholders (e.g., employees).

Beyond the CEO dark triad, we explored managerial capital as a mediator, because theoretical arguments (e.g., Palmer et al., 2020) and empirical modeling (e.g., Reina et al., 2014) suggested that a mediation process might better explain the CEO dark triad influences on performance. We focused on the TMT, with managerial capital reflecting the effectiveness of a firm’s management leadership (Yang and Lin, 2009). Direct effects were as expected, with the CEO dark triad directly reducing managerial capital, suggesting that the offensive personalities associated with the CEO dark triad do erode the strength and even composition of the TMT, which aligns with previous meta-analytic findings (Albertini and Berger-Remy, 2019). Our study extends the understanding of CEO dark personalities and suggests that they reduce the effectiveness of TMT because individuals in the TMT are likely to be most exposed to these offensive personalities, ultimately eroding trust and the TMT’s confidence in their CEO.

Interestingly, managerial capital did partially mediate the direct effect of the CEO dark triad on organizational performance, although the direct effect of the CEO dark triad was still significant. This suggests that not only does managerial capital positively shape organizational performance but CEOs with strong dark personalities are still able to shape the nature of their firm (specifically detrimentally). While managerial capital positively influenced breakthrough sales, as expected, there was no mediating effect on the CEO dark triad’s positive influence on breakthrough sales. This highlights a unique finding. The CEO dark triad can positively influence breakthrough sales and this effect is not impacted by the strength of managerial capabilities. This reinforces the calls made by Smith et al. (2018) for a greater exploration of CEO dark personalities. We suggest that the ‘show-person’ nature of a CEO with high levels of the dark triad can not only directly benefit breakthrough sales, but this effect seems to occur irrespective of managerial capital, which does have a positive effect. This is despite the literature arguing that the TMT should mediate the detrimental effects of the CEO (Palmer et al., 2020). This implies that the CEO dark triad appears to genuinely benefit the performance of breakthrough sales, supporting Smith et al. (2018).

These findings have important implications for organizational ethics, which often argue that the CEO’s dark triad can encourage unethical behaviors (Harrison et al., 2018; Chen et al., 2021, 2022). The present study meets Islam’s (2020) call for greater focus on personality within firms and highlights the challenge for ethical behavior scholars, especially since these findings challenge individual-level studies where perceptions of the CEO’s dark triad are detrimental to behaviors (Chen et al., 2021). How can firm boards be encouraged to not hire dark triad CEOs when there are potential market benefits? This is critical to understand, otherwise, such CEOs are emboldened to continue their behavior, which, here, is found to build breakthrough sales at the expense of workforce performance. However, the moderated mediation effect does provide further insights that especially challenge the potential “benefits” of a dark triad CEO.

Two-way interactions show that when firms report CEOs with high levels of the dark triad, they can maintain high levels of managerial capital and organizational performance when they operate in fiercely competitive environments. This supports our argument that firms in such environments face strong pressure to react, to be decisive, and to overcome external pressures. However, firms operating in low competitive rivalry environments do provide greater opportunity for the CEO’s offensive personality to be viewed as such, which exerts adverse internal reputational effects on the firm. We also suggested that the moderated mediation effect might counter any potential “benefit’ of a CEO dark triad, with the indirect effect weakening as competitive rivalry increased, and this was supported. Given the direct and positive effect of managerial capital—which aligns with theories on TMT factors that can disrupt the leader’s dark personality (Reina et al., 2014; Palmer et al., 2020)—we expected competitive rivalry to act as a boundary condition, which was also supported.

We also highlight an important finding from the moderated mediation models. Despite the CEO dark triad being directly related to both performance outcomes, in opposite directions, we find that the indirect effect was negative and weakened as competitive rivalry strengthened. Here, the indirect effect of the CEO dark triad on breakthrough sales becomes negative, opposite to the direct effects. This reinforces the arguments made by Smith et al. (2018) regarding exploring CEO dark personalities in more complex ways. Overall, we find that, in the context of competitive rivalry (moderator) and managerial capital (mediator), the indirect effects of the CEO dark triad become universally detrimental, counter to the otherwise positive direct effects on breakthrough sales. For ethical scholars, this provides evidence that while a dark triad CEO might “appear” beneficial, there are likely complex relationships at play, which ultimately reduce these effects to losses. This might highlight that while the CEO dark triad appears to be beneficial to performance, the otherwise largely detrimental effects found in the literature (e.g., O’Boyle et al., 2012) hold when more sophisticated tests are explored, such as moderated mediation. Here, the addition of a valued internal resource (TMT) and an external environment (competitive rivalry) teases apart the initially positive effects of the CEO dark triad on breakthrough sales, and instead reveal a more traditional and expected detrimental effect.

### Implications and future research

The implications of the present study are that firm performance might be positively influenced by the CEO dark triad, at in terms of toward breakthrough sales. Despite strong evidence that the dark triad is detrimental to employee performance (O’Boyle et al., 2012), the present study suggested that the characteristics of the CEO’s dark personality might reflect positively on customers and drive sales. While this is beneficial, there is also evidence that the CEO can still cause division and detrimental effects regarding both managerial capital and organizational performance. Thus, the CEO’s dark triad erodes the confidence and ability of the TMT to do their work, perhaps overriding resource allocation decisions that might be at odds with the CEO’s grandiose ideas. Overall, we contribute to the literature by showing both the positive and negative effects of the CEO dark triad, and showing that firm performance is shaped by CEOs through a firm’s managerial capital, which aligns with the TMT (Palmer et al., 2020). Finally, the role of context regarding competitive rivalry further highlights the complexity of understanding CEO dark triad effects and should encourage further exploration of context.

Likely, boards of directors might already be aware of these issues with such CEOs, even if they are not specifically aware of the terminology. In the New Zealand context, where employee protections are strong, it might not be as simple as “firing” the CEO. However, managing an exit might be a real option if the CEO is creating major disruptions within a firm. While encouraging CEOs high on these dimensions to “move along” might be useful, we acknowledge that their confidence and persona might make them easily “hireable” for new CEO roles. In a similar fashion, this does not also stop a firm from replacing one dark triad CEO with another. Our findings suggest that tapping into TMTs to gain insights into any new CEO appointments might be especially useful.

Research implications include more exploration of the CEO dark triad to better understand the effects on firm performance and indeed, whether our dual-edge performance effects hold. Qualitative research on how firms manage and/or expedite the exit of CEOs with high levels of the dark triad would be valuable. Indeed, mixed methods might prove especially insightful for understanding CEO dark triad effects. Following this up with interviews to ascertain *how* effects are materialized would also be useful. Similarly, understanding how those immediately below the CEO (i.e., TMT members) cope and manage CEOs with high levels of the dark triad would be insightful. Future research might explore other mediators around the TMT and also test the multi-level theoretical model of Palmer et al. (2020). For example, Wales et al. (2013) linked the CEO’s dark triad with entrepreneurial orientation, which might be a useful mediator. The present findings on moderated mediation should encourage researchers to explore these relationships further. In addition, other moderators and indeed moderators in combination (i.e., moderated-moderated mediation) might also uncover interesting insights.

### Limitations

A limitation of the present study is that the data is single-sourced. However, we followed suggestions made by Podsakoff et al. (2003) for minimizing CMB. This included having the key components of our study in different sections of the survey and spacing these with individual-focused questions (specific to the manager) to separate the focus of study constructs. While Haar et al. (2014) argue that alternative CFA model tests provide strong confidence in the constructs being distinct from each other and thus not conflated by CMB, we also followed Podsakoff et al. (2003) in terms of *post hoc* testing. We undertook the procedure described by Lindell and Whitney (2001), where we conducted a partial correlation while controlling for a construct unrelated to the relationships studied. We controlled for management positions (1 = executive, 2 = senior manager, and 3 = manager) and this new correlation showed no change in strength, which suggests that issues related to CMB are not evident. Finally, Monte Carlo simulations performed by Evans (1985) found that, in the presence of significant moderation effects, issues regarding CMB are rare. The present study found several moderation and moderated-mediation effects, which reinforce the overall argument that the influence of CMB is minimal. One limitation is the focus on New Zealand firms only, and thus future studies might expand the focus to include US and European firms to broaden the generalizability of the findings. Overall, we had a large manager sample across a broad range of industries and firm sizes, providing confidence in the generalizability of our findings.

## Conclusion

The present study explored the potential of the CEO dark triad positively influencing firm performance and provided a more nuanced understanding of relationships by exploring mediator, moderator, and moderated mediation. In doing so, we have uncovered evidence that CEOs with high levels of the dark triad can benefit a firm on one performance indicator (breakthrough sales). However, the evidence also suggests that this does not stop them from detrimentally impacting managerial capital and organizational performance, indicating that such leadership comes at a cost. We also found that the environmental context plays a largely beneficial role, bringing out the best in dark triad CEOs and also acting as a boundary condition, minimizing the indirect effect of the CEO’s dark triad as competitive rivalry strengthens. As a result of this study, we have a stronger understanding of how this dark personality impacts the way firms operate.

## Data availability statement

The raw data supporting the conclusions of this article will be made available by the authors, without undue reservation.

## Ethics statement

The studies involving human participants were reviewed and approved by Auckland University of Technology Ethics Committee (ref: 17/189, SfTI New Zealand Innovation Survey). Written informed consent for participation was not required for this study in accordance with the national legislation and the institutional requirements.

## Author contributions

JH contributed to conceptualization, data collection and analysis, and writing. The first draft of the manuscript was written by JH and KJ. Both authors commented on previous versions of the manuscript and read and approved the final manuscript.

## Funding

This study was funded by the New Zealand National Science Challenge: Science for Technological Innovation [*Kia kotahi mai - Te Ao Pūtaiao me Te Ao Hangarau*].

## Conflict of interest

The authors declare that the research was conducted in the absence of any commercial or financial relationships that could be construed as a potential conflict of interest.

## Publisher’s note

All claims expressed in this article are solely those of the authors and do not necessarily represent those of their affiliated organizations, or those of the publisher, the editors and the reviewers. Any product that may be evaluated in this article, or claim that may be made by its manufacturer, is not guaranteed or endorsed by the publisher.
